# Golden Mean and Proportion in Dental Esthetics after Orthodontic Treatments: An In Vivo Study

**DOI:** 10.3390/dj10120235

**Published:** 2022-12-12

**Authors:** Patrizia Lucchi, Giulia Fortini, Giorgia Preo, Antonio Gracco, Alberto De Stefani, Giovanni Bruno

**Affiliations:** 1 Department of Neuroscience, School of Dentistry, University of Padua, Via Giustiniani 2, 35121 Padova, Italy; 2 Department of Orthodontics, University of Cagliari, Policlinico Universitario, 09042 Monserrato, Italy; 3 Department of Pharmacological Sciences, University of Padua, Via Marzolo 5, 35131 Padova, Italy

**Keywords:** golden mean, golden proportion, dental esthetics, orthodontics

## Abstract

Purpose: The definition of the golden ratio was established around the sixth century BC; Levin and Snow developed specific theories applicable in dentistry, which apply the golden proportion rule with the intention of reproducing a perfect smile. This study analyzed the literature and assessed whether these concepts remain valid and applicable in clinical practice, evaluating the theories with a group of patients followed by an experienced orthodontic team. Methods: This study was retrospectively performed on 400 patients (241 females and 159 males) who underwent orthodontic treatments. The analysis was conducted on intraoral frontal photos, both pre-treatment and post-orthodontic treatment, to observe if there was a statistically significant difference in the tooth display according to the golden mean and golden proportion theories. Results: The canine at the end of the orthodontic treatment had a greater visibility than that proposed by Levin and Snow. Conclusions: This study revealed how these theories could be considered in certain respects, but nowadays are not totally valid and applicable to the clinical reality. Snow’s theory appears to be more consistent with the clinical findings than Levin’s theory.

## 1. Introduction

Smile analyses and designs, especially in the last decade, have become key elements in orthodontic diagnoses and treatment planning; in facial esthetics, an attractive and balanced smile is a crucial element and represents a primary goal of modern orthodontics.

There are several parameters to consider when evaluating the esthetics of a smile and these presuppose that there are many aspects to be taken into consideration for a complete dental treatment plan. Many authors, deepening the different dental disciplines, have undertaken research to try to define the parameters and protocols useful for the reproduction of the perfect smile.

Esthetic considerations generally reflect subjective criteria; the esthetic appearance of a smile mainly depends on what a professional perceives as beautiful. For this reason, the definition of scientific guidelines for dental treatments could potentially improve and standardize the results [1]. Studies in the literature underline that the analysis of the most pleasant smiles has shown that reproducible principles can be applied to improve dental esthetics [1].

The size, shape, and shade of the maxillary anterior teeth are essential for both dental and facial esthetics [2,3,4].

Many theories have been formulated regarding the evaluation of dental proportions; the concept of the “golden proportion” has often been offered as a cornerstone of smile design theories [1,4].

Lombardi [1,5] was the first to find a correlation between the golden ratio and the teeth and then Levin [1,6] applied this proportion in dental esthetics, defining the “golden proportion” (GP) theory. Ward [1,7] studied the “recurrent esthetic dental proportion” (RED). Snow [1,8] devised the golden percentage or “golden mean” (GM).

The concept of a “golden” or “divine” proportion was probably coined by Phidias (hence the term “phi” to indicate it) and has been used over the centuries as a parameter for evaluating harmonic esthetic relationships in the various fields of art, sculpture, painting, and architecture. The golden proportion is based on the theory that there is a relationship between mathematics and the beauty of nature [9].

The main purpose of this research work was to assess if the proportional parameters of the smile design theories could be taken into consideration to plan an orthodontic treatment and, by sampling pre- and post-treatment patients, to verify how effective and applicable these really were.

In this research, GP and GM theories were analyzed and applied to evaluate the dimensional relationships existing between the upper frontal dental elements (from the upper right canine to the upper left canine) according to a frontal view of the occlusion to verify whether the golden proportions were respected in a group of orthodontic patients. The null hypothesis of this study was that the theories based on the golden proportion were not supported by the clinical cases; the working hypothesis was that the golden proportion was respected.

## 2. Materials and Methods

A retrospective study was conducted analyzing 400 patients from an orthodontic private practice in Trento, Italy. The analysis was based on a sample of 241 female and 159 male patients, with an average age of 14 years and 9 months (standard deviation of 3 years and 2 months). 

The number of subjects included in our sample was significant as the literature we consulted to carry out our research took a smaller number of subjects into account; studies evaluating the relationship between the golden proportion/shape of the teeth and other characteristics of patients took as a reference a number of patients ranging from 48 to 384 [10,11,12,13,14,15,16,17,18].

The analysis was conducted using intraoral frontal photos, both pre-treatment and post-orthodontic treatment, to observe if there was a statistically significant difference in the tooth display.

The sample was selected from a photographic archive based on the following inclusion criteria:

- Caucasian origin of the patient.
- Natural permanent dentition in the upper frontal sector (from canine to canine).
- Availability of intraoral photos before and after orthodontic treatments.

Patients were not categorized based on the skeletal parameters or arch shape.

Patient photos were taken with a digital reflex camera (Nikon N90) with 60 mm optics and processed post-production with Adobe Photoshop 8.0.

All photographs were subsequently inserted into a presentation in a Keynote format.

The study exclusion criteria were as follows:

- Presence of reconstructions/prostheses in the frontal sector (from canine to canine).
- Presence of agenesis and microdontism.
- Crowding of the upper arch greater than 4 mm.

The exclusion criteria made it possible to select in the study only patients with a natural and average size of teeth. Severe dental crowding would have made it difficult to evaluate the cases and would have distorted the collection of the pre-treatment data from the photos.

The same measurements were performed in the sample at the beginning and the end of orthodontic treatments to evaluate if there was a difference between the two groups and whether the golden proportions were respected in a group of subjects with a harmonious smile at the end of a therapy carried out by an expert team.

Assessments were also performed to identify the inter-arch differences between the right and left sides of the same patient before and after orthodontic treatments.

**Figure 1:**
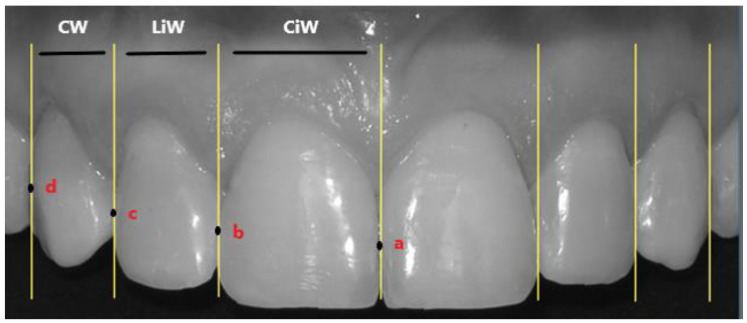
In this photo, the most important points for the evaluation of the studied measurements are highlighted. CiW (central incisor width) represents the value of the distance between point *a* and point *b* (that is to say, the width of the upper central incisor), LiW (lateral incisor width) represents the value of the distance between point *b* and point *c*, and CW (canine width) represents the value of the distance between point *c* and point *d*.

The following perpendicular reference lines were drawn for each intraoral photo (Figure 1):

- Inter-incisive line;
- Tangent lines to the two most distal points of the maximum equator of the central incisors (points *a* and *b*);
- Tangent lines to the two most distal points of the maximum equator of the lateral incisors (points *b* and *c*);
- Tangent lines to the most vestibular points of the visible surface of the canine (point *d*).

For each photo, a grid was constructed, as shown in Figure 1; in this way, the measurements of the data acquired from the study were obtained.

**Figure 2:**
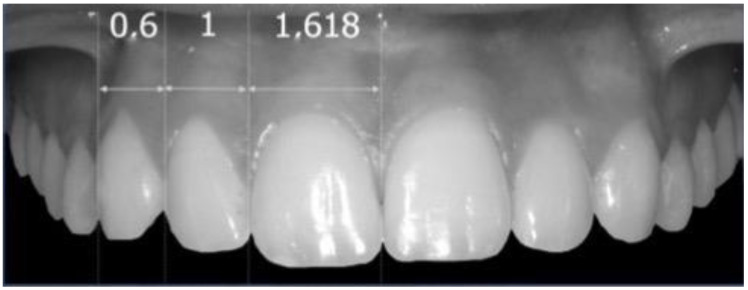
Representative image of GP theory.

**Figure 3:**
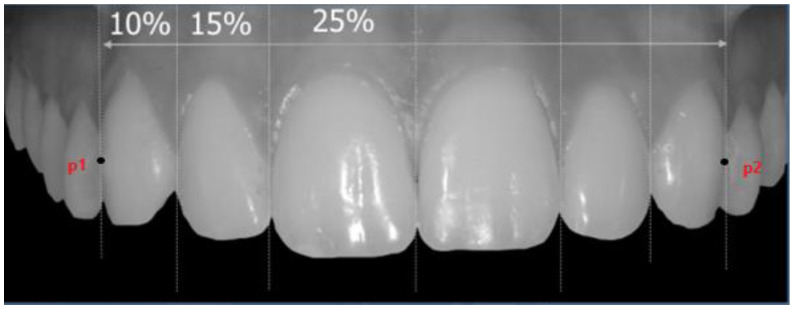
Representative image of GM theory. The distance between points *p1* and *p2* represents the inter-canine distance.

The linear distances between the traced axes were measured and proportioned to each other following the schemes of Levin and Snow:

- According to Levin (GP theory), when considering the frontal view of a smile, the width of the upper lateral incisor (LiW) has a golden proportion to the width of the upper central incisor (CiW). Assuming that the LiW has a value of 1, the CiW should measure 1.618 and the upper canine (CW) should ideally assume a value of 0.6 [6,16] (Figure 2).
- According to Snow (GM theory), when considering the frontal view of a smile, the CiW should represent 25% of the distance between the reference external tangents to the upper canines (distance between point *p*1 and *p*2; Figure 3). According to this theory, the LiW represents 15% and the CW 10% [8] (Figure 3).

Subsequently, the data collected were evaluated from a descriptive point of view to observe whether the proportions of our sample corresponded with the GP–GM theories or differed from them. A second evaluation using the Student’s t-test for paired data was performed to search for any differences between the pre-treatment and post-treatment data. The statistical analysis was performed with R 3.5 (R Foundation for Statistical Computing, Vienna, Austria). All the patient data were treated anonymously and no ethical requirements were needed as it was a retrospective analysis. 

## 3. Results

Given the size of the sample, the results for each individual patient are not shown below.

For the group evaluation, the averages of the sample measurements are reported below and are divided according to the theories of Levin (Table 1) and Snow (Table 2), both pre- and post-treatment. The statistical significance was assessed using a *p*-value threshold of 0.05.

## 4. Discussion

The definition of the golden ratio was established by Phidias around the sixth century BC; the concept of a “golden” or “divine” section has been used over the centuries as an esthetic evaluation parameter in art, sculpture, and architecture. It has been taken up by medicine over time as an attempt to correlate science with beauty. In mathematics, two quantities are in the golden ratio if their ratio is the same as the ratio of their sum to the larger of the two quantities. The ratio between the values is indicated as “ɸ” and is approximately 1.6180. This number, over time, has been attributed the value of beauty and harmony, so much to seek it and recreate it as a standard of beauty. 

In 1973, Lombardi [5] was the first author to suggest the use of the golden ratio in dentistry [1], believing that the LiW, CiW, and CW are repeated according to certain proportions. According to his “repeated ratio” theory, the ratio between the CiW and LiW should consistently progress in an anteroposterior sense. Levin [1,6] then applied this proportion in dental esthetics, developing GP theory in 1978. According to this theory, from a frontal view of an occlusion, the LiW should be 0.618 of the CiW and 0.618 of the CW [1]. According to this theory, the LiW should be 62% of the CiW; consequently, the CW should represent 62% of the LiW.

Snow [1,8] stated in 1999 that the concept of golden proportion is useful in developing a smile in which symmetry and proportion are dominant. His GM theory believes that the CiW is 25% of the upper inter-canine width according to the frontal view of a smile. Consequently, the LiW represents 15% and the CW 10%.

According to George [19], the golden ratio is considered to be a reliable predictor for determining the width of the maxillary central incisors [2].

However, these theories are not supported by several authors [1,10,17,20]; on the contrary, Preston [1,20], following a large-scale study in 1993, stated that the golden ratio was not observed in most smiles, noting that the proportionality described by Levin only occurred in 17% of the patients [20] and that none of the canines were in a golden proportion to the maxillary lateral incisors [1]. Instead, he believed that the average LiW was 66% of the CiW and that the CW was 55% of the CiW, according to the frontal view of a smile [1].

In 2001, Ward [7] pointed out that when the golden ratio is used, the lateral incisor appears too narrow and the resulting canine is not sufficiently visible. In the same year he defined the “recurring esthetic dental proportion” (RED) [1,17,18], which defines that it is very important to base assessments according to the relationship between the teeth and the facial proportion. 

More recently, other authors have posited that the theories referring to the golden proportions are not efficient in the dental field; several scientific studies [7,16,17,21] did not observe these proportions in most patients in the general population.

A recent review [1] revealed that there is currently no clear evidence in the literature to support the existence of the golden proportion in most harmonious and natural smiles, believing that compliance with these rules in dental therapies is irrelevant. It argues that, on the contrary, evaluations of the ethnic, cultural, and esthetic origins of the face are more important in the evaluation of the smiles of patients.

The observation of these differences can be related to many factors. Several studies have found that ethnicity and gender can be an influence in this sense [1,10,11,12,13,22]; in other studies, no correlation was found between the dental morphology and sex [1,10,11,12,13,22,23,24,25]. Other studies have found a correlation between the shape of the tooth and the sex and age of the patient [26,27,28].

A more practical and clinical analysis shows that, objectively, a smile and its width are related to many other aspects, including the arch shape, the presence of buccal corridors, skeletal divergence, and the shape of the face. All these aspects influence the dental golden proportions to an extent. A narrower arch shape corresponds with a less visible exposure of the dental elements; studies have shown that the inter-canine distance varies among arch shapes with a significant difference in the upper arch [29,30]. 

The buccal corridors are spaces that have interested clinicians as an important aspect of the esthetics of a smile [31]; their presence allows the natural progression of a smile to be highlighted. Frush and Fisher observed that the absence of buccal corridors led to an unnatural look [31,32]. Moore found evidence supporting that a smaller buccal corridor is related to a more attractive smile [31,33]. 

Although several researchers have stated that buccal corridors make no difference to the evaluation of smiles [31,34,35,36], most researchers state that the size of the corridor of a smile is critical to its esthetic value [31,33,37,38,39,40], and that this is mainly perceived by orthodontists [31].

Skeletal divergence is also important and is generally related to the muscle conformation and face shape of patients; knowledge of the relationship between dental and skeletal characteristics helps both in the diagnostic evaluation and in the planning of dental treatments [41]. The skeletal facial height is related not only to the shape of the face, but also to the shape of the upper dental arch; a hyper-divergence is correlated to a decrease in the transverse width of the upper arch whereas hypodivergence is generally correlated to more developed upper arches [41,42,43].

A few theories argue that there is an association between the face shape and tooth shape, believing that thinner faces are mostly related to a narrower and elongated tooth shape whereas hypodivergent faces are associated with a taurodontic tooth shape [14,26,44]. A facial analysis is believed to be important in the planning of prosthetic cases and conservative dentistry; a few authors have argued that the face configuration can be analyzed as a guide to realize the tooth shape for a customized patient smile [45]. Other studies have revisited this theory to verify its validity; to date, it is not supported by all researchers [26,27,46] despite being a theory that guides the prosthetic approach in edentulous patients in many cases [46].

Even the morphology of individual dental elements can affect the evaluation of the proportionality proposed by Levin and Snow. The presence of an altered Bolton Index or of widespread microdontism, as demonstrated by Mirabella [15], can determine a negative contribution to the proportionality of a smile.

Another aspect that the authors did not consider in this study, but one that is surely relevant in the perception of the esthetic proportion of dental elements, is tooth color and shade [47]. 

The analysis of the results of this study highlighted several concepts that agreed with the reports by Levin and Snow [6,8], but there were also conflicting conclusions.

The justification for the difference in the proportionality between the right and left sides of the patients evaluated in the study was largely due to the photographic evaluation. As the intraoral photographs were all taken at a dental unit by turning the patient slightly to their right, it is likely that there was not a perfect perpendicularity in the documentation collection.

A measurement of the dental casts would probably have significantly reduced this difference. However, it would have been necessary to use a measurement system that envisaged the use of reference grids; this method would certainly have been more complex than the one used and, given the large number of cases analyzed, the study would have required more time.

A further study will be undertaken to evaluate how much the individual dental rotations of canines and laterals can influence these proportions because 79 out of the 400 patients presented dental rotations in the photographic analysis (for a total of 102 rotated elements). It should be specified that the rotations did not lead to a crowding in the arch greater than 4 mm, which was in accordance with the inclusion criteria used for the selection of the sample.

The best concordance of the statistical data between Snow’s proportional ratios concerning Levin’s golden proportions was probably found in the different quantifications of the numerical data. Levin proposed criteria that were most affected by the measurements whereas Snow related the individual values to a global inter-canine distance.

According to the data collected and evaluating the proportionality model proposed by Levin, it was possible to observe how the CW increased regarding the golden proportion, assuming an average value of about 0.92 pre-treatment and 0.85 post-treatment (considering an average of the values between the right and left proportion) compared with the value of 0.6 assumed by the GP theory. This difference assumed a statistically significant value and indicated that the projection decreased following the treatment.

The CiW, on the other hand, assumed values that were not statistically significantly different from the values proposed by Levin (an average of 1.60 in the pre-treatment and an average of 1.57 in the post-treatment compared with 1.68 supported by the golden theory proportion).

More representative and stable was the mode of representation of the frontal sector used by Snow, which was closest to the data collected in our study. However, a lower percentage representation of the central incisors could be observed compared with that proposed by Snow, both in the pre- and in the post-treatments (mean: 22.5% pre-treatment; mean: 22.78% post-treatment) as well as a higher CW both pre- and post-treatment (average: 13% pre-treatment; average: 12.56% post-treatment). In this case, the small percentage variations could be correlated to the rotations present in the sample.

Snow’s assessment of the golden mean showed that only the proportions of the lateral incisors significantly changed (from an average value of 14.53% to 14.67%) as well as the canines (from an average value of 12.98% to 12.56%). There were no statistically significant changes in the central incisors between the pre- and post-treatments.

In the analysis of the data, it could also be stated that the projection increased after treatments for the lateral incisors whereas it decreased for the canines. An explanation for this could lie in the orthodontic operative steps. 

## 5. Conclusions

The authors conducted research on 400 patients whose dental characteristics were evaluated according to GP and GM theories. The results showed that these models were still valid in many aspects, but needed to be updated in others. The proportions proposed by Snow appeared to be more respected in our sample, but the superior canine in the frontal view of a smile appeared to be more represented, in a mesiodistal sense, than what had been proposed by Levin and Snow.

The significant differences in the analysis of the data between pre- and post-orthodontic treatments provided a starting point to reflect on the further developments of this work concerning the relationships between dental proportions and gender, ethnicity, and skeletal patterns. 

## Figures and Tables

**Table 1:** Sample values calculated by applying GP theory (Levin).

Pre-Treatment Analysis		Dental Elements					
		Right Upper Canine	Right Upper Lateral Incisor	Right Upper Central Incisor	Left Upper Central Incisor	Left Upper Lateral Incisor	Left Upper Canine
	Mean	0.915	1	1.572	1.631	1	0.940
	SD	0.176	/	0.208	0.754	/	0.658
Post-Treatment Analysis	Mean	0.844	1	1.485	1.646	1	0.876
	SD	0.006	/	0.170	0.141	/	0.098
*p*-Value		0.000006	/	0.000711	0.004660	/	0.026841
		***	/	***	***	/	*

SD: standard deviation. *: *p*-value < 0.05, ***: *p*-value < 0.01.

**Table 2:** Sample values calculated by applying GM theory (Snow).

Pre-Treatment Analysis		Dental Elements					
		Right Upper Canine	Right Upper Lateral Incisor	Right Upper Central Incisor	Left Upper Central Incisor	Left Upper Lateral Incisor	Left Upper Canine
	Mean (%)	13.09	14.52	22.57	22.43	14.53	12.87
	SD	1.16	1.43	1.38	1.91	1.78	1.66
Post-Treatment Analysis	Mean (%)	12.96	15.41	22.75	22.81	13.92	12.15
	SD	0.410	1.569	0.292	0.381	1.429	0.123
*p*-Value		0.000063	0.003546	0.102258	0.648437	0.000003	0.014120
		***	***	ns	ns	***	*

SD: standard deviation. The values reported as the *p*-value represent the evaluation of the pre- and post-treatment statistical data using the Student’s *t*-test. A statistical evaluation of the pre- and post-treatment data was then performed using the Student’s *t*-test and the statistical significance was assessed using a *p*-value threshold of 0.05. *: *p*-value < 0.05, ***: *p*-value < 0.01.

## Data Availability

Not applicable.
